# Must we remain blind to undergraduate medical ethics education in Africa? A cross-sectional study of Nigerian medical students

**DOI:** 10.1186/s12910-017-0229-2

**Published:** 2017-12-08

**Authors:** Onochie Okoye, Daniel Nwachukwu, Ferdinand C. Maduka-Okafor

**Affiliations:** 1 Department of Ophthalmology, College of Medicine, University of Nigeria, Ituku-Ozalla, Enugu, Enugu State Nigeria; 2 Department of Physiology, University of Nigeria, Enugu Campus, Enugu, Nigeria

**Keywords:** Medical ethics, Undergraduates, Curriculum, Education, Dilemma, Nigeria

## Abstract

**Background:**

As the practice of medicine inevitably raises both ethical and legal issues, it had been recommended since 1999 that medical ethics and human rights be taught at every medical school. Most Nigerian medical schools still lack a formal undergraduate medical ethics curriculum. Medical education remains largely focused on traditional medical science components, leaving the medical students to develop medical ethical decision-making skills and moral attitudes passively within institutions noted for relatively strong paternalistic traditions.

In conducting a needs assessment for developing a curriculum germane to the Nigerian society, and by extension most of Sub-Saharan Africa, this study determined the views of Nigerian medical students on medical ethics education, ethical issues related to the doctor-patient relationship and the ethical/professional dilemmas they are confronted with.

**Methods:**

Using self-administered 63-item structured questionnaires, a cross-sectional survey of the final year medical students of the University of Nigeria was conducted in July 2015.Using the Statistical Package for the Social Sciences software (SPSS Version 17), frequency counts and percentages were generated.

**Results:**

The sample included 100 males (71.4%) and 40 females (28.6%), with the respective mean (SD) age being 24.6(5.61) and 21.8 (6.38) years. Only 35.7% were satisfied with their medical ethics knowledge, and 97.9% indicated that medical ethics should be taught formally. Only 8.6% had never witnessed a medical teacher act unethically. The dilemmas of poor communication between physicians and patients, and the provision of sub-standard care were reported highest for being encountered ‘often’. A majority (60.7%) indicated that “a doctor should do his best always, irrespective of the patient’s wishes”. No significant difference in responses across gender was noted.

**Conclusion:**

There is a strong desire by the contemporary Nigerian medical student for medical ethics education. Their lack of exposure in medical ethics in an ethically challenging environment suggest a dire need for the development of an appropriate medical ethics curriculum for them and the provision of an ethically conducive learning environment.

## Background

The practice of medicine in the modern era is beset with unprecedented challenges in virtually all cultures and societies [1].Medical students and resident doctors in training today are learning in an ethical environment that is increasingly complex. It is thus becoming imperative that the policies and processes being put in place to address this and other issues should guarantee a climate that is favourable and conducive to the ethical development of future medical practitioners [2].

With this changing role of medicine and the growing expectations by patients, the content and delivery of medical curricula also have to change [3].As part of this drive, it was recognised that medical ethics education should be accorded a greater formal presence in the medical curriculum [4]. By 1999, the World Medical Association’s (WMA) 51st General Assembly had recommended that medical ethics and human rights be taught at every medical school as obligatory and examined parts of the medical curriculum; a resolution which was subsequently reinforced and revised in October 2015 [5] in recognition of the fact that the practice of good medicine inevitably raises both ethical and legal issues and demands an understanding of both [6].

In Nigeria, however, medical education remains largely focused on traditional clinical and basic medical science components, leaving students to develop moral attitudes passively through observation and intuition [7]; a situation that may not be too far from what it is obtainable in most of Sub-Saharan Africa [8–10]. There is evidence that the ‘hidden’ curriculum confers a powerful influence on the ethical and professional development of the students as they observe the norms, behaviours and interactions in their learning environments [4]; with the doctor-patient relationship being the predominant interaction.

There is still no formal medical ethics curriculum being seriously implemented in many Nigerian medical schools. Though medical ethics may exist as part of the curriculum in some of these schools, no curricular time is assigned. The apparent lack of institutional interest may be related to the lack of trained, capable faculty and the paucity of ethics educational resources and other infrastructures in these schools. Currently, there is no indigenous and locally available standardized undergraduate medical ethics text in the list of recommended textbooks within the Nigerian university system. More often than not, the only exposures for the medical students may be the informal ethics mentions by their supervising doctors in the course of their training, the occasional talks during their orientation programs or special events organised by the state chapter of the Nigerian Medical Association and also, media or internet reports on medico-ethical issues. Finally, on graduation, the newly inducted doctors are given the Code of Medical Ethics booklets together with their professional registration licenses but this still does not necessarily translate to a commensurate level of interest and knowledge [11].

There is no uniformity in what the students learn through the ‘hidden’ or ‘informal’ curricula, and there is no way of ensuring that a minimum standard is being met. The relative decline of ethical/professional standards, coupled with the deterioration of the doctor-patient relationship and the increasing criticisms of physicians by the public, have heightened the need for undergraduate medical ethics education. Current global best medical practices attach great importance to the concepts of informed consent and shared decision making, and these students need to be educated on the various models of the doctor-patient relationship; of which the universally-accepted ideal remains the shared decision-making model. There is therefore a need to ensure that the medical schools can vouch for a minimum level of medical ethics knowledge, medico-ethical competencies, interactional skills set and, even the character of their graduates.

However, teaching medical ethics as merely a scientific body of knowledge may be dangerous if it misses the individualistic and communal perceptions of morality and ethics within their unique socio-economic, cultural, and geographic contexts [12]. In developed countries where it is being taught widely, medical ethics in its present form, remains largely rooted in western culture [10].Hence, the curriculum of medical ethics should be tailored to the local needs and peculiarities of the society where it is taught. In order to formulate a medical ethics curriculum germane to the six-year undergraduate medical degree program in Nigerian universities specifically, and most of Sub-Saharan Africa generally, educational needs assessment is imperative. As a preliminary step, this study was undertaken to determine the views of Nigerian medical students on medical ethics education and medico- ethical issues related to the doctor-patient relationship, as well as the ethical/professional dilemmas with which they may be confronted.

## Methods

Using self-administered 63-itemstructured questionnaires, a cross-sectional survey of the final year medical students of the College of Medicine, University of Nigeria was conducted in July 2015 after a scheduled lecture. Eighty-one items were initially generated from literature review [12–17] and emergent themes from previously conducted focus group discussions with pre-registration house-officers and junior resident doctors employed in the affiliated teaching hospital. In order to ensure some degree of validity, these items were consequently reviewed for relevance, appropriateness, difficulty and ambiguity by 3 senior medical educators/clinicians. The 63-item questionnaire was finally adopted, after 18 items were rejected. No prior information about the test items being tested was provided to them, and the questionnaires were retrieved immediately after completion at the venue; both measures aimed at minimising response bias. However, to reduce any possible misinterpretations of the test items arising from their brevity, the respondents were encouraged to seek clarifications on any item from the investigators. Such clarifications were provided openly to the entire sample very time.

The questionnaire had 4 major sections: the first section dealt with the socio-demographic data namely sex, age and religious affiliation; the second section with their views on 15 items regarding medical ethics education and their experience/witnessing of ethical dilemmas; the third section with their views on 10 clinical ethical issues related to the doctor-patient relationship and the fourth section on the frequency with which they witnessed selected 35 ethical/professional dilemmas. In section 2, the students were asked the following: if they had any previous formal exposure to medical ethics, if they were satisfied with their current knowledge of medical ethics, if medical ethics should be taught formally to medical students, if medical ethics should be a mandatory course and be assessed in medical schools, if medical ethics is best taught by medical doctors, if medical ethics is important for professional practice, if medical ethics is just ‘common sense’, if every medical student should be interested in medical ethics, if they had experienced an ethical dilemma, and if they had witnessed any teacher acting unethically. In addition, they were also required to indicate the following: their main type of formal medical ethics exposure, their main source of medical ethics information, the best time for introducing the teaching of medical ethics to medical students, the duration of formal ethics teaching required and the most important quality expected in medical ethics teachers. All the items required single responses to options provided.For instance, if the options provided for any item were ‘Yes’, ‘No’ and ‘Not sure’, or ‘often’, ‘sometimes’, ‘never’ and ‘not sure’, the respondent was only required to select one response. The responses for the third section were ranked on a scale of 1(Disagree), 2(not sure), and 3(Agree) and for the fourth section, options provided were ‘often’, ‘sometimes’ and ‘never’. For section 3, the respondents were required to indicate their level of agreement with the perception statements provided. For three of the items, there were ‘correct’ answers we adopted i.e. bordering on the importance of confidentiality, consent being necessary for every procedure and the doctor doing his best for the patient always. For some other more contentious items, we sought to simply determine the baseline pattern of responses i.e. disclosure of medical errors to patients by their doctors. For sections 3 and 4,only single responses on all the test items were required, as well.

Data were then entered into an Excel database and using the Statistical Package for Social Sciences program version 17.0(SPSS inc), frequency counts and percentages were generated over the range of responses. The Chi-square test was used to determine the levels of significance, with *P* values <0.05 being considered statistically significant. The study was approved by the Health Research Ethics Committee of the University of Nigeria Teaching Hospital, Ituku-ozalla, Enugu, Nigeria.

## Results

The sample included 100males (71.4%) and 40 females(28.6%), with the respective mean (SD) age being 24.6(5.61) years and 21.8(6.38) years; a response rate of 91.5%.The majority of these undergraduate medical students(62.1%) were aged between 21 and 25 years. All the respondents were Christians, with the majority (44.3%) belonging to the Roman catholic denomination.

Though the University does not run a formal training program in medical ethics, a majority of the students stated that they had been formally exposed in variable degrees to medical ethics within their undergraduate education; with a sizeable proportion of these students not being able to specify the type of formal exposure obtained and none being capable of specifying clearly the title of any medical ethics textbook read or didactic lecture ever received in school (Table 1).Among the 83 who stated that they had been formally taught medical ethics, a greater segment referred to the traditional one-time pre-clinical orientation talk for new clinical students as the main type of formal medical ethics teaching received (Table 1). An overwhelming majority of the 140 respondents (97.9%) were of the view that medical ethics should be introduced and taught formally as a course (Table 2).A majority of the students (47.4%) were in support of commencing the formal teaching of medical ethics in the clinical years, namely 3rd MBBS class(Pathology and Pharmacology as core subjects), 4th MBBS class (Paediatrics, Obstetrics & Gynaecology, Community Medicine as core subjects) and 5th MBBS class(Medicine and Surgery as core subjects). Only 8.6% had never witnessed a medical teacher act unethically, in their own perception (Table 1).Tables 1 and 2 present data on their views regarding the 15 test items in section 2 of the study questionnaire; mainly concerned with their exposure to medical ethics education, perception of medical ethics education and witnessing of ethical dilemmas. The responses of the students were also sought on some perception statements relevant to the doctor-patient relationship (Table 3). Their responses with respect to ‘agree’ and ‘disagree’ options were evenly distributed on three of the perception statements investigated, namely, “Children should never be treated without the consent of their parents/guardians”, “Patients should always be informed of every medical error” and “Close relatives should always be told about the patients’ condition” (Table 3). Table 3 presents data relevant to the 10 test items in section 3 of the study questionnaire. The majority (60.7%) were of the opinion that “a doctor should do his best always, irrespective of the patient’s wishes” (Table 3). The dilemmas of poor communication between physicians and patients, and the provision of sub-standard care were reported highest with respect to being encountered ‘often’ (Table 4). Table 4 presents data from section 4 of the study questionnaire on the 35 test items regarding various ethical/professional dilemmas which were witnessed/encountered by these students. Comparisons done between the male and female respondents regarding their responses did not demonstrate any statistically significant difference.

**Table 1:** Distribution of the 140 Students Regarding their Exposure to Medical Ethics(ME) Education and Ethical Dilemmas

Issue	Response	Number	%
Previous formal exposure to ME	Yes	83	59.3
	No	48	34.3
	Not Sure	9	6.4
Main Type of ME exposure	Orientation talk	38	45.8
	Clinical teachings Special NMA lecture Not specified	9 5 31	10.8 6.0 37.4
Satisfied with ME knowledge	Yes	50	35.7
	No Not sure	65 25	46.4 17.9
Main ME information source	Media/internet	76	54.3
	Medical books Medical journals Clinical teachings Ethics textbooks Miscellaneous Didactic lectures	44 11 1 0 8 0	31.4 7.9 0.7 0 5.7 0
Ethical dilemma experience	Often Sometimes Never Not sure	18 73 24 25	12.9 52.1 17.1 17.9
Teachers’ unethical conduct	Often Sometimes Never Not sure	35 74 12 19	25 52.9 8.6 13.6

*Key: NMA-Nigerian Medical Association*

*Often-at least weekly*

*Sometimes—monthly/less frequently/seldom*

*Never—no incident experienced*

**Table 2:** Distribution of the 140 Medical Students Regarding their views on other Issues relevant to their need for Medical Ethics (ME) Education

Issue	Response	Number	%
ME should be taught formally to medical students	Yes No Not sure	137 2 1	97.9 1.4 0.7
ME should be a mandatory course and be assessed	Yes No Not sure	123 11 6	87.9 7.9 4.3
Best time to introduce the ME course	1st session 2nd MB class 3rd MB class 4th MB class 5th MB class Not sure	3 14 31 23 11 55	2.2 10.2 22.6 16.8 8 40.1
Duration of ME teaching needed	<one year One year >one year Continuous Not sure	22 16 29 41 32	15.7 11.4 20.7 29.3 22.9
The most important quality in ME teachers	Character Competence Any of the 2 Others Not sure	97 18 11 2 12	69.3 12.9 7.9 1.4 8.6
ME is best taught by medical doctors	Yes No Not sure	129 2 9	92.1 1.4 6.4
ME is important for professional practice	Yes No Not sure	132 6 2	94.3 4.3 1.4
ME is just ‘common sense’	Yes No Not sure	41 85 14	29.3 60.7 10
Every student should be interested in ME	Yes No Not sure	130 7 3	92.9 5 2.1

**Table 3:** Medical students responses to statements concerning the doctor-patient relationship (*n* = 140)

Perception statement	Agree. No(%)	Dis-agree. No(%)	Not sure. No(%)
Patient’s wishes must be respected always	84(60)	41(29.3)	15(10.7)
Doctor should do his best irrespective of patient’s wishes	85(60.7)	37(26.4)	18(12.9)
Confidentiality is not important in medical practice	16(11.4)	119(85)	5(3.6)
Consent is only necessary for surgeries and not for medical procedures or laboratory tests	12(8.6)	126(90)	2(1.4)
Close relatives should always be told about the patient’s true medical condition or status	51(36.4)	59(42.1)	30(21.4)
Patients should always be informed by their doctor of every medical error	55(39.3)	58(41.4)	27(19.3)
Children should never be treated without the consent of their parents/guardians	60(42.9)	60(42.9)	20(14.3)
Patients refusing treatment on religious grounds should be referred elsewhere	80(57.1)	38(27.1)	22(15.7)
If patient wishes to die, doctors should assist to facilitate this	10(7.1)	113(80.7)	17(12.1)
Termination of pregnancy should be offered to any willing female who requests it	20(14.3)	104(74.3)	16(11.4)

**Table 4:** Frequency with which the 140 students witnessed dilemmas related to issues of Decision-making for treatment, Justice, Communication, Professionalism and Student-Specific Concerns

Dilemma	Often(%)	Sometimes (%)	Never(%)
Poor Doctor/Patient communication Substandard care being provided Doctor/Patient care conflicts Non-adherence to treatment Decisional capacity concerns Religious beliefs Doctors going beyond professional obligations Disrespect to colleagues, students Doctors encouraging queue-jumping in clinic Doctors disrespect to patients Student keeping quiet over teachers conduct Doctors treating despite poor prognosis Students not allowed to examine patients Student learning on patients unsupervised Breaking bad news Gratuitous story-telling about patients Shortage of drugs/consumables Student learning on patients without consent Patients as research subjects Informed consent issues Student doing intimate physical examinations Uncertainty over role in health team Dealing with difficult patients Medical/surgical error Questionable use of resources Discriminatory care of patients Abuse /neglect of vulnerable persons Student Compromising one’s ethical standards Privacy/confidentiality issues Medical error disclosure Blood transfusion concerns Truth-telling vs deception Surrogate decision making Paternity test decisions Do not resuscitate orders	64(45.7) 64(45.7) 48(43.3) *59(42.1)* 55(39.3) 55(39.3) 54(38.6) 54(38.6) 54(38.6) 53(37.9) 51(36.4) *51(36.4)* 50(35.7) 50(35.7) 42(30) 40(28.6) 40(28.6) 39(27.9) 37(26.4) 36(25.7) 36(25.7) 36(25.7) 35(25) 34(24.3) 29(20.7) 26(18.6) 26(18.6) 24(17.1) 23(16.4) 21(15) 19(13.6) 19(13.6) 14(10) 4(2.9) 3(2.1)	63(45) 68(48.6) 85(60.7) 62(44.3) 79(56.4) 67(47.9) 74(52.9) 70(50) 72(51.4) 66(47.1) 75(53.6) 73(52.1) 66(47.1) 72(51.4) 79(56.4) 66(47.1) 69(49.3) 78(55.7) 70(50) 82(58.6) 69(49.3) 81(57.9) 69(49.3) 80(57.1) 43(30.7) 69(49.3) 73(52.1) 53(37.9) 72(51.4) 57(40.7) 104(74.3) 52(37.1) 55(39.3) 31(22.1) 19(13.6)	13(9.3) 8(5.7) 7(5) 19(13.6) 6(4.3) 18(12.9) 12(8.6) 16(11.4) 14(10) 21(15) 14(10) 16(11.4) 24(17.1) 18(12.9) 19(13.6) 34(24.3) 31(22.1) 23(16.4) 33(23.6) 22(15.7) 35(25) 23(16.4) 36(25.7) 26(18.6) 68(48.6) 45(29.3) 41(29.3) 63(45) 45(32.1) 62(44.3) 17(12.1) 69(49.3) 71(50.7) 105(75) 118(84.3)

*Key: often-at least weekly*

*sometimes—monthly or less frequently/seldom*

*never-no incident experienced*

## Discussion

Medical ethics education is widely considered to have come of age [18].With ethics and professionalism being globally recognised as an integral component of the primary medical degree curriculum, it is presumed that early exposure of medical students may be beneficial in establishing a solid base in ethics [19].On this note, it seems that there is consensus that mainstreaming and formalising ethics education in the medical school curriculum increases knowledge and confidence in medical ethics among medical students [20].

Our study had a high response rate whereas, several related studies conducted in various parts of the world had recorded response rates ranging from 46.5% to 96% [7, 13, 15, 17, 20–23]; which may merely be a reflection of the levels of interest displayed by the various study populations or the types of study procedures adopted.Our study demonstrates a strong desire by these students for formal medical ethics education against the backdrop of the observed low level of their medical ethics education (Tables 1 and 2). This was further buttressed by the finding that the majority of the students were either not satisfied or uncertain about their knowledge in medical ethics. In another related study among final year medical students from the University of Ibadan, Nigeria, most students(80.5%) responded that they did not receive enough training in medical ethics and 85.9% believed that formal ethics education would be worthwhile [7].Interestingly, a study conducted among Nigerian medical doctors demonstrated that 80% of the respondents admitted having some previous medical ethics education as undergraduates; with the median duration of exposure being three hours [11].However,86% of these doctors deemed their exposure as grossly inadequate; a finding which is hardly surprising because most medical schools in Nigeria still have no formal medical ethics program. Several studies carried out among students and even physicians in many developing countries have also shown that the majority of their respondents expressed strong agreement on the importance of undergraduate medical ethics education [15, 19–21], even when being implemented minimally or inconsistently and with many of them making a case for properly structured medical ethics program across all levels of medical education.

The importance attached to a particular curricular element or activity may also be assessed by its position in the school calendar or the amount of time allocated. It is noteworthy that a greater proportion of our respondents expressed uncertainty over the ideal time for the introduction of medical ethics teaching or a preference for it to be introduced in the clinical phases of the medical program (Table 2). This may be an indication of their apparent perception of the existing medical curriculum as being already over-loaded, or their lack of information regarding the extent and scope of the proposed medical ethics curriculum. Some authors however opine that the first exposure of medical students to medical ethics should be within their first two years of school, and that it should be taught step by step throughout pre-clinical and clinical education [24, 25]. Since medical ethics is so important to medical education and practice, it should be vertically and horizontally integrated into the medical curriculum (starting preferably with a foundation course); a situation that may be in line with the views of a greater proportion of the students that the formal learning of medical ethics, once commenced,should continue for the entire duration of their medical program.

For the developing countries like Nigeria with peculiar challenges over malpractice and medical negligence, medical ethics education may actually be a process towards achieving the hitherto elusive regulation of medical practice [10].At a minimum, a more practical, pragmatic and measurable goal for the undergraduate medical ethics program may then be to endow our students with a set of cognitive and behavioural skills for ethical reasoning that will allow them to recognise ethical dilemmas in clinical practice and research, and equip them to reach socio-culturally sensitive, practical and ethically-defensible solutions to those dilemmas. Pertinent to any serious discussions about teaching medical ethics to undergraduate medical students in our setting will of course be consideration of issues such as determining the content areas, the specific objectives/learning outcomes, the format for organising the contents, educational strategies according to Harden et al.’s model [4],teaching methods and assessment methods. The scope of this present study did not cover these important aspects with our respondents.

Significant gaps still remain in our understanding of the ways in which ethics curricula may contribute to improvements in the physician’s ability to identify and handle ethical problems. Over the years, there has been a raging controversy on determining the concept of ethical sensitivity among medical students [26], though some posit that extensive curricular intervention in ethics teaching is effective for raising knowledge and confidence of medical students in facing ethical challenges [27]. In our study, the majority stated that they had experienced or witnessed ethical dilemmas in the course of their undergraduate program whereas 17.9% were not sure of having witnessed these challenges (Table 1); a possible pointer to their lack of confidence/ability in recognising moral issues. This particular study has demonstrated that these students commonly experience a wide range of ethical and professional challenges in the course of their medical education and clinical rotation (Table 4); bordering on decision-making over treatment/care, communication, professionalism issues, justice issues and student-specific issues as described in other studies [28, 29].The findings also bring to the fore the overlap between ethics and professionalism in healthcare; with medical professionalism being relevant to medical ethics as long as it concerns those attributes which foster health practitioners’ abilities to recognise, interrogate, and to enact the ethical duties they possess in their roles [30]. The majority of the medical students stated that they had also witnessed a medical teacher acting unethically in the course of their program (Table 1).It must be stressed, however, that these students were merely reporting perceived unethical behaviour. They may not have had complete information or better insight into those witnessed acts which may really not have been unethical. Such clinical dilemmas can shape medical students’ ethical and professional development and these experiences constitute an informal or ‘hidden’ ethics curriculum [31]. These clinical teachers who act as negative role models, especially those who show unethical behaviour towards patients, are the most frequently cited problematic aspects of the medical curriculum [31].If the gap between what is taught formally and what is practised or witnessed is wide, the message sent to the students becomes diluted, distorted and mis-interpreted; considering the fact that students sometimes also look for humanistic qualities in their teachers. Given the sensitive and controversial nature of many ethical issues in medical practice and education, the provision of an environment conducive to students’ raising questions and expressing their opinions is imperative to promote genuine discourse on such issues; many of which may be largely unknown to their medical teachers. Without positive role-modelling and guidance, these students should not be expected to recognise, reason, acknowledge and act ethically through all ethical dilemmas confronting them all the time.

The range of their responses to the perception statements on doctor-patient relationship is indicative of the variation in the students’ ethical sensitivity, depending on their knowledge, beliefs,values, experiences and interactions with their patients/attending doctors. The tension existing between the principles of respect for autonomy and beneficence (non-maleficence) and the tendency towards being paternalistic in the course of clinical decision-making is highlighted by the pattern of responses on the items addressing refusal of treatment on religious grounds, euthanasia/physician-assisted suicide and wilful termination of pregnancies; with socio-cultural and religious perspectives probably exerting their influences on these choices (Table 3).This could also be indicative of the lack of ethics training on the part of these students, who can only learn from the prevailing attitudes and practices of their medical teachers, most of whom apparently belong to the paternalistic traditions. In ethical dilemmas where conflicting values, preferences, principles, risks and benefits play out, a more structured analytical approach is imperative in achieving justifiable resolution. Without adequate ethics education, a vicious cycle of paternalism will invariably continue to dominate in the doctor-patient relationships in our settings.

For any effective medical ethics teaching program in our medical schools, efforts are needed to remove the impediments in the clinical educational environment, which may hinder the proper moral and professional development of these medical students [32]. A supportive system that is committed to the promotion of appropriate professional behaviour within our medical schools and the affiliated teaching/specialist hospitals remains the key to achieve this. The future of medical ethics education may not have to rely solely on formal curricular development but also in the creation of an ethical environment in which students can learn and honestly ask questions courageously, health workers can practise professionally and patients can receive humane care [33]. As part of the needs assessment, the views of other stakeholders such as the medical teachers, institutional and departmental heads, patients and other health care personnel may prove invaluable in developing an effective and appropriate medical ethics program for our medical schools. Inputs from the Medical and Dental Council of Nigeria (regulatory agency for medical practice), the National Universities Commission and the Nigerian Medical Association would also be essential to developing and implementing such curricula, not just for undergraduates but across all levels of medical education in the country.

## Conclusion

This study has highlighted the strong desire by the contemporary Nigerian medical student for formal medical ethics education. Their lack of exposure in medical ethics in an ethically/professionally challenging environment highlights the need for the development of an appropriate medical ethics curriculum and the provision of an ethically conducive learning climate for positive role-modelling by the teachers and cultivation of moral courage by the students. In keeping in line with global best practices, the medical student in Nigeria, and by extension Africa, has definitely come of age to benefit from formal medical ethics teaching programs, and as such medical schools can no longer afford to remain blind to this development.
